# Investigating the relationship between domestic violence with substance abuse and suicide resilience in mothers with disabled children

**DOI:** 10.3389/fpubh.2023.1223896

**Published:** 2023-08-17

**Authors:** Fateme Mohammadi, Majid Barati, Seyed Reza Borzou, Elahe Ezati, Khadejeh Mohammadi, Zahra Mohammadi, Salman Khazaei, Seyedeh Zahra Masoumi

**Affiliations:** ^1^Department of Nursing, School of Nursing and Midwifery, Chronic Diseases (Home Care) Research Center and Autism Spectrum Disorders Research Center, Hamadan University of Medical Sciences, Hamadan, Iran; ^2^Department of Public Health, School of Health, Autism Spectrum Disorders Research Center, Hamadan University of Medical Sciences, Hamadan, Iran; ^3^Department of Medical Surgical Nursing, Department of Nursing, School of Nursing and Midwifery, Chronic Diseases (Homecare) Research Center, Hamadan University of Medical Sciences, Hamadan, Iran; ^4^Department of Public Health, Asadabad School of Medical Sciences, Asadabad, Iran; ^5^Poya1 Special School, District 2 Education, Isfahan, Iran; ^6^Education of Isfahan Province, Isfahan, Iran; ^7^Health Sciences Research Center, Health Sciences and Technology Research Institute, Hamadan University of Medical Science, Hamadan, Iran; ^8^Department of Midwifery, School of Nursing and Midwifery, Mother and Child Care Research Center, Hamadan University of Medical Sciences, Hamadan, Iran

**Keywords:** domestic violence, substance abuse, suicide resilience, mothers, disability, children

## Abstract

**Introduction:**

Mothers with disabled children are among the most critical groups exposed to domestic violence. Although domestic violence strongly affects these mothers’ physical and mental health, it subsequently affects their drug addiction and resilience to suicide. Based on this, it is crucial to investigate domestic violence, drug addiction, and resilience against suicide in mothers with disabled children. This study investigated the relationship between domestic violence, substance dependence, and resilience against suicide in mothers with disabled children in Iranian society.

**Methods:**

From January to April 2023, a cross-sectional study was conducted in central and western Iran with the participation of 267 mothers with disabled children. The mothers of disabled children were selected through convenience and snowball sampling. Then they completed questionnaires included domestic violence, substance dependence and resilience against suicide. The collected data were analyzed using SPSS version 22 with descriptive statistics, such as prevalence, percentage, mean, and standard deviation, and expository measurements, including ANOVA, independent *t*, and regression tests.

**Results:**

The study revealed that there was a strong direct correlation between domestic violence and substance abuse (*r* = 0.89, *p* < 0.001), as well as a strong indirect correlation between domestic violence and suicide resilience (*r* = −0.90, *p* < 0.001). Additionally, substance abuse and suicide resilience were negatively correlated (*r* = −0.93, *p* < 0.001). Other variables, such as the severity of children’s disability, education, financial status, and the fathers’ involvement, were predictors of domestic violence, accounting for 73.28% of the variance.

**Conclusion:**

Mothers with disabled children reported moderate levels of domestic violence, which strongly impacts their physical and mental well-being, leading to drug dependency and suicide. So, it is essential to implement comprehensive planning and provide extensive support to reduce domestic violence against them. By doing so, we can enhance their physical and mental health and ultimately improve their overall quality of life.

## Introduction

Domestic violence is one of the most challenging social problems that lead to serious physical injuries and psychological problems in people (1, 2). The prevalence of domestic violence is primarily influenced by the cultural norms and values a family and society uphold. However, vulnerable groups are more exposed to domestic violence across all communities (1). Among these vulnerable groups, women are particularly at risk of domestic violence (3). The World Health Organization states that any Violent gender-related behavior that causes physical, sexual, and psychological harm accompanied by threats, coercion, or absolute deprivation of women’s authority and freedom overtly or covertly is considered as a violence against women (4). Studies indicate that violence against women is a prevalent issue worldwide, with incidence rates ranging from 24 to 71 percent in different regions (5, 6). In the United States, 30% of women are physically abused by their husbands, and their husbands even beat 40% of these women (6). Of the women who have been victims of domestic violence, 38.5% have been killed by their husbands. In France, one out of every ten women suffers from domestic violence, and every year about 100,000 women are sexually abused (7). The World Health Organization’s report highlights that domestic violence affects a notable proportion of women globally, with estimates ranging from 27.8 to 32.2 percent. The prevalence is exceptionally high in African countries (45.6%), Southeast Asia (40.2%), and the Eastern Mediterranean (8). The prevalence of violence against women in Iran varies significantly depending on cultural background. According to a study, Zahedan has the lowest rate of violence against women at 5.4%, while Tehran has the highest rate at 95% (9). The prevalence and reporting of domestic violence against women in Iran are influenced by cultural norms that vary among ethnic groups (9, 10). However, factors such as social class, substance abuse, and the low income of husbands also contribute to domestic violence (8, 9). Research suggests that women with lower education levels, unemployed women, and those with disabled children or fertility issues are particularly vulnerable to violence within Iranian culture (11–14). In Iran, there are approximately 78,000 children with exceptional needs, including those with physical-motor, mental, behavioral-emotional, autism, vision, and hearing disorders (5, 15). These study show almost one in three children with disabilities worldwide has experienced violence (16). Studies have shown that disabled children are 3.8 times more likely to be neglected or physically abused, 3.1 times more likely to be sexually abused and 3.9 times more likely to be emotionally abused. In fact, findings show that 31% of disabled children suffer abuse compared with 9% of the non-disabled child population. Further to this, disabled children are also at a higher risk of experiencing multiple abuses and of enduring multiple episodes of abuse (17, 18). Accurate statistics on domestic violence against children and adolescents, especially children and adolescents with disabilities, are not available. But the results of the study Vameghi et al. (19) asserted, 22.8 percent of the high school students in Tehran were exposed to physical domestic violence. The mothers of these children are primarily responsible for their care, which often results in significant mental and emotional strain. Sadly, many of these women also experience physical and emotional abuse from their husbands and families due to the birth of a disabled child (7, 17). The research found that children with disability are twice as likely to have a mother hospitalized due to a domestic violence assault (20). There is no accurate information about the amount of domestic violence against mothers of disabled children in Iran. Based on this, the need for more investigation and studies is well felt (17, 20). However, the domestic violence can lead to chronic headaches, feelings of helplessness, lack of self-confidence, anxiety, depression, drug abuse, and even suicide among these mothers (12, 21, 22).

Substance abuse pertains to the excessive and compulsive consumption of drugs, psychoactive substances, and narcotics in a manner that harms the user or others (8, 23). Although drug addiction and abuse predominantly affect men in Iran (24), recent statistics indicate that the number of women addicted to drugs has increased fourfold in the past decade, with 9% affected (25). Numerous studies suggest that women may become dependent on drugs due to various factors, including pressure from spouses or friends, a desire to escape reality, poverty, and domestic violence (22, 25, 26). Furthermore, Mothers of disabled and exceptional children experience significant psychological stress when they are subjected to violence by their spouses and relatives due to the birth of their disabled child. This stress severely affects their mental health and confidence (21). These mothers may turn to drugs to cope with the pressures they face, particularly if they feel unable to protect themselves and their children from violence (12, 15, 27). Unfortunately, Numerous studies have shown that survivors of domestic violence are more likely to contemplate suicide and lack resilience against it (28–30). An international study has demonstrated that sexual violence is one of the most constant risk factors in reducing resilience against suicide among women (29). These studies suggest that mental health policies and services should recognize the relationship between domestic violence and resilience against suicide in women and consider the effect of cultural and social factors in understanding domestic violence against women and its associated adverse effects (28, 29). Despite some quantitative and qualitative studies investigating domestic violence and its impact on women’s physical and mental health, domestic violence in mothers of disabled and vulnerable children and its association with substance abuse and resilience against suicide in these mothers have not been examined yet. Therefore, given the aforementioned issues, it is essential to conduct a study to investigate the relationship between domestic violence, substance abuse, and resilience against suicide in mothers of disabled and vulnerable children in Iranian society.

## Methods

### Study design and setting

This cross-sectional study utilized the strengthening reporting of observational studies in epidemiology statement (STROBE) for observational research from January to April 2023. The study aims to evaluate domestic violence, substance abuse, and suicide resilience in mothers with disabled children and assess the relationship between domestic violence, substance abuse, and suicide resilience in these mothers.

### Participants and sampling

The study followed the methodology of Afkhamzadeh et al. (11) with *β* of 80% and *α* of 0.05, accounting for a 10% dropout rate to determine the sample size. As a result, 267 mothers of disabled children were selected through convenience and snowball sampling from six welfare centers and three charity centers in Iran’s western and central regions. The inclusion criteria were literacy, willingness to participate, having at least one disabled child (physical-motor, mental, blind, deaf, developmental-behavioral), at least one year since the definitive diagnosis of the child’s illness, and no history of other physical or mental disorders. Mothers with disabled children who failed to answer more than half of the items on their questionnaires or did not return their questionnaires were excluded. The participants were asked to complete the questionnaires, including demographic characteristics, domestic violence against women, suicide resilience, and Leeds dependence. The majority of the questionnaires (71.64%) were gathered entirely in April. 232 of the subjects completed questionnaires. Thus, the response rate was 86.89%. The participants’ reasons for not being completed in this study were losing the questionnaires and not being motivated.

### Questionnaire

#### Demographic information questionnaire

The questionnaire included the child’s age and gender, number of family members, children’s disability, level of education, and financial situation.

#### Domestic violence scale

The Domestic Violence Questionnaire was developed by Tabrizi et al. (31). It consists of four subscales, including psychological, economic, physical, and sexual, which evaluate violence against women with 60 questions. These questions are scored on a 5-point Likert scale ranging from never (0) to always (5). A score between 0 to 60 indicates low domestic violence, a score between 60 to 120 indicates moderate domestic violence and a score above 120 indicates high domestic violence against women. In 2020, Tabrizi reported the content validity of this questionnaire in the Iranian community. The reliability of this questionnaire was also calculated with a Cronbach’s alpha coefficient of 0.83, indicating a high-reliability level (31).

#### Leeds dependence questionnaire

In 1994, Resick et al. designed a questionnaire to measure dependence on various substances. The questionnaire consists of 10 questions that are scored on a four-point Likert scale (0–3). Scores less than 10 indicate low dependence, scores between 10–22 indicate moderate dependence and scores greater than 22 indicate high dependence. In 2016, Habibi et al. reported that the content validity of this questionnaire is both convergent and divergent. Additionally, the reliability of this questionnaire has been calculated with Cronbach’s alpha coefficient 0.90 (32).

#### Suicide resilience questionnair

The suicide resilience questionnaire was created by Osman et al. (33) to develop a multidimensional tool for suicide that could be used in various studies. The scale aims to measure an individual’s perceived ability to cope with suicidal thoughts, the availability of external resources, and the individual’s assessment of their ability to deal with adverse events. The questionnaire consists of 25 questions that assess internal protective dimensions, external protective dimensions, and emotional stability The Likert scoring method is used in this questionnaire, with response options ranging from “completely agree” to “completely disagree”, assigning a score of 1 to 6, respectively. Therefore, the participant’s score in this questionnaire will range from 25 to 150, with higher scores indicating greater resilience to suicide. The reliability of the questionnaire and its measures has been reported between 0.90 and 0.95. The validity of this tool has also been calculated using various methods, all of which were satisfactory (34).

#### Statistical methods

This study analyzed the collected data with SPSS software version 22. For this purpose, descriptive statistics (frequency, percentage, mean, and standard deviation) were used. ANOVA and independent *t*-tests were also used to investigate the relationship between domestic violence with substance abuse and suicide resilience and demographic information in mothers with disabled children. The significance level was considered *p* < 0.05. Then the demographic variables, suicide resilience and substance abuse with domestic violence (*p* < 0.25), were entered into the multiple linear regression model with a backward strategy. Before performing various linear regressions, the researcher evaluated hypotheses, including data normality, variance homogeneity, and residual independence.

#### Ethical considerations

The studies involving human participants were reviewed and approved by the Asadabad of school of medical science (IR.ASAUMS.REC.1402.014) with code of projeh (134). The researcher introduced herself and explained the study’s goals at the outset, assuring participants that all information would remain confidential and that they could withdraw from the study at any point. After receiving adequate information about the study, all participants provided written informed consent.

## Results

### Demographic information

The study included mothers of children with disabilities, whose ages ranged from 23 to 54 years, with an average age of 39.32 ± 2.31. Of the participants, 129 (55.60%) had completed secondary school, and most were self-employed, 98 (42.24%). Additionally, most mothers had sons (121, 52.16%), and the most common type of disability among the children was mental (81, 34.91%). Also, a significant relationship was found between domestic violence and the type and severity of the child’s disability, as well as the mother’s education, financial status, and the father’s addiction (Table 1).

**Table 1:** The participants’ demographic characteristics and domestic violence score in mothers of children with disabled.

Demographic variables		Number (%)	Death anxiety means ±SD	*p*-value
Mother’s age (year)	23–33	73 (31.46)	86.32 ± 2.13	0.901**
	34–44	104 (44.82)	85.74 ± 2.41	
	44–54	55 (23.71)	85.12 ± 2.16	
Mother’s education	Illiterate	10 (4.32)	103.62 ± 2.31	0.015**
	Primary	44 (18.96)	98.11 ± 2.86	
	Diploma	129 (55.61)	83.95 ± 2.05	
	Bachelor	27 (11.63)	41.41 ± 2.24	
	Master’s degree and higher	22 (9.48)	22.13 ± 2.57	
Mother’s job	Self-employed	98 (42.24)	78.32 ± 2.14	0.017**
	Employee	34 (14.65)	43.98 ± 2.68	
	Housewife	100 (43.10)	94.58 ± 2.14	
Number of children	1	87 (37.50)	70.64 ± 2.39	0.832**
	2	104 (44.82)	69.08 ± 2.52	
	3 and more	41 (17.67)	69.04 ± 2.13	
Sex of children	Boy	121 (52.16)	82.43 ± 2.21	0.832*
	Girl	111 (47.84)	82.57 ± 2.54	
Children’s age	3–6	68 (29.31)	83.58 ± 2.39	0.858**
	7-11	91 (39.22)	86.31 ± 2.14	
	12–14	73 (31.46)	81.95 ± 2.62	
Kind of disability	Physical-motor	81 (34.91)	97.98 ± 2.53	0.013**
	Mental	98 (42.24)	102.58 ± 2.27	
	Blind	10 (4.32)	42.53 ± 2.21	
	Deaf	11 (4.74)	47.44 ± 2.34	
	Developmental-behavioral	32 (13.79)	61.21 ± 2.27	
Severity of disease	Mild	44 (18.96)	71.38 ± 2.13	0.011**
	Moderate	124 (54.44)	94.23 ± 2.53	
	Sevier	64 (27.58)	99.48 ± 2.76	
Financial status	Lose than<100$	73 (31.46)	105.78 ± 2.42	0.019**
	100-200$	100 (43.10)	98.64 ± 2.97	
	>200$	59 (25.43)	89.48 ± 2.76	
Father’s addiction	Only cigarettes	100 (43.10)	65.38 ± 2.72	0.019**
	Heroin + cigarettes	41 (17.67)	88.63 ± 2.64	
	*Salvia divinorum* + cigarettes	55 (23.71)	99.48 ± 2.13	
	Opium + cigarettes	36 (15.51)	106.38 ± 2.422	

*Independent *t*-test. **ANOVA test.

#### Domestic violence, substance abuse, and suicide resilience in mothers with disabled children

Mothers of children with disabilities participating in this study reported a domestic violence score of 106.89 ± 3.15, a substance abuse score of 19.09 ± 2.63, and a suicide resilience score of 89.08 ± 2.20 (Table 2).

**Table 2:** The means and standard deviations of the participants’ domestic violence, suicide resilience and substance dependence.

Variable	dimensions	Mean ± SD (each dimension)	Mean ± SD (total)
Domestic violence	Psychological	146.78 ± 3.12	106.89 ± 3.15
	Physical	103.47 ± 3.21	
	Sexual	79.58 ± 3.07	
	Economic	97.74 ± 3.22	
Suicide resilience	Internal protective	78.17 ± 2.32	89.08 ± 2.20
	External protective	82.32 ± 2.16	
	Emotional stability	75.07 ± 2.14	
Substance dependence	Without dimension	19.09 ± 2.63	19.09 ± 2.63

#### The relationship between domestic violence, substance abuse, and suicide resilience in mothers with disabled children

Findings in this study revealed that there is a strong and direct correlation between domestic violence and substance abuse (*r* = 0.89, *p* < 0.001) and a strong and indirect correlation between domestic violence and suicide resilience (*r* = −0.90, *p* < 0.001). Also, there is a strong and indirect correlation between substance abuse and suicide resilience (*r* = −0.93, *p* < 0.001) in mothers with disabled children (Table 3).

**Table 3:** Relationship between domestic violence, substance dependence, suicide resilience in mothers of children with disabled.

Domestic violence	Substance abuse	*r* = 0.89	*p* < 0.001
Domestic violence	Suicide resilience	*r* = −0.90	*p* < 0.001
Substance abuse	Suicide resilience	*r* = −0.93	*p* < 0.001

#### Predictors of domestic violence in in mothers with disabled children

The variables substance abuse suicide resilience, substance abuse, suicide resilience, kind and severity of children’s disability, education and financial status, and father’s addiction with a *p*-value of smaller than 0.25 were entered into multiple linear regressions with the backward technique. These variables remained in the model and accounted for about 73.28% of the domestic violence variance in mothers with disabled children (Table 4).

**Table 4:** The predictor variables domestic violence in chi mothers of children with disabled.

Factors		Non-standard coefficients		standard coefficients	*T*	*p*-value
		*B*	Standard error	*β*		
Substance dependence		3.32	2.11	0.56	1.57	0.001
Suicide resilience		3.11	1.78	0.62	1.74	0.001
Financial status		2.79	1.33	0.63	2.09	0.014
Severity of disease	Mild	Reference	–	–	–	–
	Moderate	2.46	1.24	0.45	1.98	0.011
	Sevier	2.43	1.23	0.43	1.97	0.010
Kind of disability	Physical-motor	Reference	–	–	–	–
	Mental	2.28	1.23	0.42	1.85	0.012
	Developmental-behavioral	2.24	1.22	0.41	1.83	0.013
	Deaf	2.20	1.19	0.38	1.84	0.015
	Blind	2.19	1.17	0.36	1.87	0.014
Mother’s education	illiterate	Reference	–	–	–	–
	Primary	1.87	1.02	0.34	1.83	0.015
	Diploma	1.85	1.04	0.33	1.77	0.016
	Bachelor	1.83	1.11	0.30	1.64	0.015
	Master’s degree and higher	1.80	1.09	0.29	1.65	0.017
mother’s job	Self-employed	Reference	–	–	–	–
	Employee	1.76	1.05	0.31	1.67	0.017
	housewife	1.72	1.03	0.28	1.66	0.019
Father’s addiction	Only cigarettes	Reference	–	–	–	–
	Heroin+ cigarettes	1.71	1.02	0.28	1.67	0.019
	*Salvia divinorum*+ cigarettes	1.68	1.01	0.26	1.66	0.017
	Opium+ cigarettes	1.67	1.02	0.25	1.63	0.019

Adjusted R2: 73.28%.

## Discussion

The present study’s findings indicate that mothers of disabled children experience moderate domestic violence and have a relatively high tendency towards substance abuse, which the high level of domestic violence has caused. These mothers report moderate resilience against suicide. Furthermore, the study’s findings demonstrate a direct and strong relationship between domestic violence and substance abuse in mothers of disabled children and a strong inverse correlation between domestic violence and resilience against suicide. On the other hand, the study’s findings indicate that substance abuse, suicide resilience, the type and severity of the children’s disability, education, and financial status, and the father’s addiction explain a high percentage of the variance in domestic violence in mothers of disabled children. Although several studies have examined domestic violence, resilience against suicide, and substance abuse in women from different cultures, no study has assessed domestic violence, resilience against suicide, and substance abuse in mothers of disabled children. Therefore, the author has used other articles that have examined domestic violence, resilience against suicide, and substance abuse in women with different conditions and diseases in various cultures to write the discussion. The average score for domestic violence among mothers of disabled children in this study was 106.89 ± 3.15, indicating that these mothers reported moderate levels of domestic violence. Although some studies suggest that violence is prevalent among disabled children, child laborers, and women, no research is currently available that specifically evaluates domestic violence in mothers of disabled children. Therefore, domestic violence has been discussed in different cultures concerning women. This study found domestic violence to be average among mothers of disabled children, with mothers of mentally disabled children reporting the highest levels of domestic violence. Additionally, there was a statistically significant correlation between domestic violence in mothers of disabled children and substance abuse, suicide resilience, the type and severity of the children’s disability, education and financial status, and the father’s addiction.

In line with this study, Kageyama et al. (35) stated that domestic violence is more prevalent in families with schizophrenic patients, with the highest rate of domestic violence (51%) reported against mothers who are severely affected by the family’s economic and cultural conditions. According to Dhunna et al.’s (36) findings, while violence against mothers is prevalent worldwide, Young Māori mothers in Aotearoa/New Zealand experience numerous violent behaviors in their families, especially from their sexual partners. Poverty, substance abuse, child and caregiver health status, racism, and social and cultural background are influential factors. Lessard et al. (37) have reported that domestic violence against mothers is prevalent and is a vital factor in disrupting their mental health and leading them toward substance abuse. Domestic violence, particularly sexual violence, has been identified as the primary cause of maternal distress. Therefore, it is essential to implement protective and preventive measures to reduce violence against mothers and improve their physical, sexual, and mental health (37). Zhang et al. (38) highlighted that fathers’ violent behaviors toward mothers are one of the most significant factors that lead to children exhibiting violent behaviors toward their mothers, including physical, behavioral, and verbal abuse. Furthermore, violent behaviors toward mothers are associated with economic status, fathers’ addiction, and mental health. Therefore, it is essential to implement appropriate policies to support mothers and reduce violent behaviors toward them in households (38). In line with the present study Amel Barez et al. (3) also stated that it is evident that violence against pregnant women is relatively high in Iran. Abusive behaviors are associated with mothers’ financial, mental health, educational, and occupational status. Pregnant women resort to strategies such as improving their situation or escaping from conditions that lead to violence to protect themselves and their infants. Policymakers need to enforce stricter laws to protect the rights of women and pregnant mothers, as well as children with disabilities, and carefully follow up on cases of violence against them and impose appropriate penalties (3).

In this study, the average score of substance dependence in mothers of disabled children was reported as 19.09 ± 2.63. It was also found that there is a strong and direct correlation between substance abuse and domestic violence, as well as a strong and indirect correlation between substance abuse and resilience against suicide in mothers of disabled children. This study is consistent with Bailey et al.’s (39) findings, which suggest that women with a history of high levels of domestic violence, stress, and anxiety are more likely to turn to drug use. Therefore, extensive and organizational support can reduce the psychological stress of these women and decrease their inclination towards drug use (39). In 2021, Kadir Shahar et al. reported high levels of physical, psychological, and sexual violence against women in Malaysia, particularly violence by sexual partners. It was noted that sexual violence against women is associated with partner addiction, low education levels, and social status. They also stated that violent behavior decreases women’s resilience and increases their tendency towards substance abuse (40). Hisasue et al. (41) also stated that domestic violence against women is relatively high, severely reducing their quality of life and causing mental distress, depression, and their tendency to use drugs, which is in line with the present study. Iruye et al. (42) also stated that although domestic violence during childhood strongly affects the psyche of women and causes disturbances in their mental health, they experience many mental and emotional tensions that sometimes cause them to turn to drugs, which is in line with the present study.

The study found that mothers of disabled children scored an average of 89.08 ± 2.20 on the resilience scale against maternal suicide. The results showed an inverse correlation between domestic violence and drug addiction with resilience against suicide. However, Nooshabadi et al. (43) reported a direct and significant relationship between domestic violence and resilience against suicide in Iranian women. Women who experienced domestic violence had a greater tendency towards suicide, which is in line with the findings of this study (43). According to Kazemi Khooban et al. (44), domestic violence against women imposes significant psychological stress on them, leading to anti-social behaviors and suicidal thoughts. Factors such as poverty, cultural weakness, and low education levels contribute to increased violence against women. Violence with mental tension and suicidal thoughts reinforce each other as a vicious cycle (44). Atadokht et al. (45) also state in their study that women with addicted husbands endure high levels of domestic violence, which severely leads to depression and suicidal tendencies. Although this finding is consistent with the present study, Atadokht et al. identified women with addicted husbands and evaluated their inclination toward suicide. However, the present study examined mothers with disabled children who often experience high levels of psychological stress due to their child’s disability, criticism from their surroundings, and the cost of their child’s care and treatment. Based on this, domestic violence against mothers with disabled children is more prevalent, and their resilience against suicide is lower (45). Kapoor, et al. (46) also stated in their study that African-American women’s resilience against suicide is inversely related to physical, emotional, and sexual abuse in childhood, and it is directly related to individual abilities, self-efficacy, and spiritual well-being. Therefore, the development of support for women and the prevention of their abuse during childhood, as well as increasing their self-confidence can increase their resilience against suicide (46). This study has investigated women’s resilience against suicide in women with a history of abuse, and although it reports average resilience against suicide in line with the current study, it was conducted in a different cultural context from Iranian society. Dorsey Holliman et al. (47) also state that social support, support of friends and family members, and religious beliefs increase the resilience of African American women veterans. Spates et al. (48) also state that the many experiences of poverty, discrimination, and low social status of black women have caused them to be able to face adverse conditions and achieve high self-concept and self-esteem, which strengthens their resilience against suicide. Although these studies also state that women’s financial conditions, attitudes and beliefs are effective on their resilience against suicide, these studies examine resilience against suicide in women with different conditions than mothers of disabled children. Therefore, resilience against suicide in these women is somewhat different from mothers of disabled children.

Based on the results of this study, it can be concluded that mothers with disabled children have experienced moderate domestic violence, which has led to increased psychological stress and decreased resilience toward suicide and substance abuse. Therefore, it is essential to plan and provide extensive support for reducing domestic violence, improving physical and mental health, and enhancing the quality of life for these mothers.

## Limitations

One of the main limitations of the current study was the non-return of questionnaires due to a lack of motivation and loss of questionnaires by mothers. Therefore, it is recommended that domestic violence, substance abuse, and resilience against suicide in mothers with disabled children in various communities and with larger sample sizes be evaluated to obtain a more accurate estimate of domestic violence against mothers with disabled children. Another limitation of this study was the collection of information only through questionnaires. Therefore, it is suggested that other data collection methods, including interviews and observations, be used in future studies. Another limitation was the presence of mediator and moderator variables, as there are many mediator and moderator variables in the assessment of domestic violence against mothers with disabled children, and this study only focused on the effects of some of them. The researchers were not able to evaluate some mediator and moderator variables including the experience of violence in the mother during childhood, the experience of violence in the father during childhood, the history of sexual abuse in mothers during childhood and adolescence. Therefore, it is suggested that the effects of other mediator and moderator variables be examined in future studie. One of the limitations of the current study was the lack of investigation into the causes of domestic violence and substance dependence in mothers with disabled children. Therefore, it is recommended that the causes of domestic violence and substance dependence in mothers with disabled children be investigated in future studies.

## Conclusion

Mothers with disabled children experience moderate domestic violence, significantly affecting their physical and mental health, resilience, and drug addiction. On the other hand, substance abuse, suicide resilience, the type and severity of the child’s disability, education and financial status, and the father’s addiction significantly impact the incidence of domestic violence against these mothers, predicting 73.28% of the variance. Therefore, welfare officials and community health advocates must minimize domestic violence against mothers with disabled children through close monitoring, frequent visits, and extensive support.

## Data availability statement

The original contributions presented in the study are included in the article/supplementary materials, further inquiries can be directed to the corresponding author.

## Ethics statement

The studies involving human participants were reviewed and approved by the Asadabad of school of medical science (IR.ASAUMS.REC.1402.014) with code of projeh (134). The participants provided their written informed consent to participate in this study.

## Author contributions

FM, SM, SK, ZM, KM, SB, MB, and EE were involved in the conception of the study and designed the study, drafted the primary manuscript, and responsible for data collection. FM, SM, MB, SB, EE, KM, ZM, and SK analyzed data. All authors contributed to the article and approved the submitted version.

## Conflict of interest

The authors declare that the research was conducted in the absence of any commercial or financial relationships that could be construed as a potential conflict of interest.

## Publisher’s note

All claims expressed in this article are solely those of the authors and do not necessarily represent those of their affiliated organizations, or those of the publisher, the editors and the reviewers. Any product that may be evaluated in this article, or claim that may be made by its manufacturer, is not guaranteed or endorsed by the publisher.
